# Local evidence on the cytomegalovirus viral load threshold for preemptive treatment is welcome, and a comment on indirect effects

**DOI:** 10.1590/2175-8239-JBN-2021-E009

**Published:** 2021-11-05

**Authors:** Lúcio R. Requião-Moura

**Affiliations:** 1 Universidade Federal de São Paulo, Departamento de Medicina, Disciplina de Nefrologia, São Paulo, SP, Brasil.; 2 Hospital Israelita Albert Einstein, Unidade de Transplante Renal, São Paulo, SP, Brasil.

Cytomegalovirus (CMV) infection is one of the most common events after kidney transplantation, with implications for clinical management, morbidity, and possibly mortality^1^^,^ 2. In the primary infection, observed in 10% of patients, the transmission occurs throughout the graft from a seropositive donor to a seronegative recipient. On the other hand, most recipients had the first contact with the virus in childhood, and reactivation occurs after the immunosuppression, when CMV-related events are divided into direct and indirect effects^4^^,^ 5. Direct effects are infection when there is evidence of CMV replication independent of symptoms and disease when infection is followed by CMV-related symptoms or laboratory changes^4^. The indirect effects observed are an increased risk of secondary infections (pneumocystis and other herpesviruses) and an increased risk of acute rejection and chronic kidney dysfunction^5^.

In this scenario, it is imperative to adopt one of two strategies to reduce the risk of CMV-related events, especially the direct effects: universal prophylaxis or preemptive treatment^4^. The first strategy is based on the use of valganciclovir for 3 or 6 months after transplantation, which seems to be associated with fewer CMV-related events, despite some disadvantages such as toxicity, late-onset CMV disease, risk of resistance, and high cost^6^. Preemptive treatment, on the other hand, is the sequential and intensive monitoring of CMV replication to detect early viral load, followed by antiviral treatment when a threshold viral load is reached before symptoms appear^4^. Since the public health system in our country does not provide valganciclovir, most transplant centers perform CMV risk reduction with preemptive treatment. However, to date, the best viral load threshold to start treatment with this strategy has not been clearly defined.

I read with great interest the article by Caurio and coworkers^7^ published in the Brazilian Journal of Nephrology. They enrolled 30 kidney transplant recipients who were transplanted at two different centers and prospectively collected 232 plasma samples to identify CMV viremia using two paired methods: pp65 antigenemia and an in-house quantitative renal time PCR. For PCR, they calibrated the results according to the 1st WHO International Standard (WHOIS) for Human Cytomegalovirus. The included patients were at low or high risk for CMV-related events after transplantation, as 40% of them had received immunological induction with anti-lymphocyte depletion antibody (antithymocyte globulin). In addition, they all received tacrolimus and mycophenolate as maintenance immunosuppression and 86.6% were CMV IgG positive before transplantation.

The milestone of results was the performance of the in-house PCR to predict CMV-related events, defined as the decision to initiate antiviral treatment in a preemptive treatment strategy, based on the pp65 antigenemia result (10 positive cells) or physician decision. Three different viral loads were evaluated, 2,750 IU/mL, 3,430 IU/mL, and 3,650 IU/mL, which achieved sensitivity for antiviral treatment of 100%, 97.1%, and 91.2%, respectively. For the value of 3,430 IU/mL (log 3.54), the ROC curve to initiate therapy was 0.93. As well pointed out by the authors in the discussion of the article, *after nearly ten years of the launch of the WHOIS, a consensual threshold for treatment of CMV has not yet been defined*. It is well known that viral load correlates with the risk of CMV-related events, particularly disease and invasive disease. However, given the wide variation among populations, risk factors, and laboratory aspects such as specimen types (whether plasma or whole blood) and assays, the third international consensus recommends that centers establish viral load thresholds for therapy or treatment endpoints. For this goal, the study appears to be successful^4^.

In the study, of 30 patients, 25 had a positive test, 11 were symptomatic, but only 3 met the criteria for CMV disease. It is important to note that the viral load in the asymptomatic patients was 3,490 IU/mL (*vs*. 15,539 in the symptomatic, P<0.001), which was closer to the value found with better performance to predict treatment (3,430 IU/mL) when 10 positive cells in pp65 antigenemia and physician decision were used as the gold standard. We must acknowledge the authors' efforts and the good quality of this evidence. However, this cut-off value cannot be extrapolated to other centers using commercial or other in-house PCR. For instance, in a previous study, David-Neto et al. investigated cut-offs for pp65 antigenemia and an in-house PCR, and the best results for predicting CMV disease were 4 positive cells and 2,000 copies/mL, respectively^8^.

Regardless of the threshold for preemptive treatment (2,000, 3,500, or 5,000 IU/mL), this strategy exposes patients to some levels of CMV replication that could be associated with the indirect CMV effects. In an elegant and prospective study, Reischig et al. demonstrated that CMV viremia ≥ 2000 copies/mL increased the probability of interstitial fibrosis and tubular atrophy in kidney allograft biopsies (OR=3.83, P=0.023)^9^. We found that CMV viremia was associated with high urinary levels of retinol-binding protein (OR = 4.62, P = 0.001), an early marker of proximal tubular damage, resulting in lower 5-year graft function (OR = 0.95, P = 0.01)^10^. On the other hand, a recent publication indicated that current clinical management should no longer consider the impact of CMV infection on long-term outcomes anymore^11^. At the moment, there is not enough evidence to reject the hypothesis of indirect effects, at least while further investigations are required to address our concerns reagrding kidney transplant recipients.
